# Noradrenergic stimulation modulates activation of extinction-related brain regions and enhances contextual extinction learning without affecting renewal

**DOI:** 10.3389/fnbeh.2015.00034

**Published:** 2015-02-19

**Authors:** Silke Lissek, Benjamin Glaubitz, Onur Güntürkün, Martin Tegenthoff

**Affiliations:** ^1^Department of Neurology, BG University Hospital Bergmannsheil, Ruhr-University BochumBochum, Germany; ^2^Faculty of Psychology, Department of Biopsychology, Institute of Cognitive Neuroscience, Ruhr-University BochumBochum, Germany

**Keywords:** atomoxetine, contextual extinction learning, fMRI, noradrenaline, renewal effect

## Abstract

Renewal in extinction learning describes the recovery of an extinguished response if the extinction context differs from the context present during acquisition and recall. Attention may have a role in contextual modulation of behavior and contribute to the renewal effect, while noradrenaline (NA) is involved in attentional processing. In this functional magnetic resonance imaging (fMRI) study we investigated the role of the noradrenergic system for behavioral and brain activation correlates of contextual extinction and renewal, with a particular focus upon hippocampus and ventromedial prefrontal cortex (PFC), which have crucial roles in processing of renewal. Healthy human volunteers received a single dose of the NA reuptake inhibitor atomoxetine prior to extinction learning. During extinction of previously acquired cue-outcome associations, cues were presented in a novel context (ABA) or in the acquisition context (AAA). In recall, all cues were again presented in the acquisition context. Atomoxetine participants (ATO) showed significantly faster extinction compared to placebo (PLAC). However, atomoxetine did not affect renewal. Hippocampal activation was higher in ATO during extinction and recall, as was ventromedial PFC activation, except for ABA recall. Moreover, ATO showed stronger recruitment of insula, anterior cingulate, and dorsolateral/orbitofrontal PFC. Across groups, cingulate, hippocampus and vmPFC activity during ABA extinction correlated with recall performance, suggesting high relevance of these regions for processing the renewal effect. In summary, the noradrenergic system appears to be involved in the modification of established associations during extinction learning and thus has a role in behavioral flexibility. The assignment of an association to a context and the subsequent decision on an adequate response, however, presumably operate largely independently of noradrenergic mechanisms.

## Introduction

Renewal in extinction learning occurs when a response acquired in a particular context and extinguished in a different, novel context, reappears during extinction recall in the context present during acquisition (Bouton and Bolles, 1979). A prototypical renewal experiment therefore consists of three phases: *acquisition* refers to learning of an association between a cue and a consequence/response in context A. In the following phase, *extinction learning*, the cue is presented in context B and no longer followed by its original consequence, which leads to extinction of the conditioned response. In the final test phase termed *extinction recall*, the cue is again presented in context A, renewing the response learned during acquisition. The renewal effect of extinction evoked by this so-called ABA design has been demonstrated in a wide variety of tasks, ranging from fear extinction learning (Bouton and King, 1983), taste aversion learning (Rosas and Bouton, 1997) and appetitive conditioning (Bouton and Peck, 1989) in rats to fear conditioning (Vansteenwegen et al., 2005, 2006) and predictive learning (Üngör and Lachnit, 2006, 2008; Lachnit et al., 2008; Lissek et al., 2013) in humans. The renewal effect in humans was recently shown to be mediated by vmPFC and hippocampus in concert (Lissek et al., 2013), with hippocampus encoding the relation between context and cue-outcome during extinction learning and vmPFC active during extinction recall to retrieve this association. The renewal effect impressively underlines the context-dependency of extinction learning. It has been suggested that this sensitivity to context is caused by the unexpected change in the cue-outcome relation occurring during extinction learning, which in turn triggers enhanced processing of environmental stimuli that correlate with this unexpected event, such as the context (Bouton, 2004; Rosas and Callejas-Aguilera, 2006). Attention is assumed to have a central role in processing the extinction context and thus in evoking a renewal effect (Darby and Pearce, 1995; Rosas and Bouton, 1997; Rosas and Callejas-Aguilera, 2006; Uengoer and Lachnit, 2012). Recent studies of human extinction learning showed that extinction learning aroused attention to the context (Nelson et al., 2013), in particular if context is relevant (Lucke et al., 2013; Rosas et al., 2013). These results are in line with the finding that attention towards stimuli with high informational value is stronger than towards those with low informational value, i.e., irrelevant stimuli (George and Pearce, 2012).

A brain region crucial for attentional processing as well as for extinction learning and retrieval is the medial prefrontal cortex (PFC). Medial PFC is involved in attention (Bussey et al., 1997; Kahn et al., 2012), particularly in mediating shifts of attention (Owen et al., 1991, 1993; Birrell and Brown, 2000). In addition, ventromedial PFC has a prominent function in contextual extinction learning (Maren et al., 2013) and the renewal effect (Lissek et al., 2013). Consistent with its role for attention, the medial PFC is a target region for the noradrenergic system. Noradrenaline (NA) in the forebrain is provided by the locus coeruleus, which projects to prefrontal regions, in particular infralimbic medial PFC, to the hippocampus and the amygdala (Jones et al., 1977; Loughlin et al., 1986) Therefore, manipulating NA in this region may modulate contextual extinction learning which requires attentional processing.

Noradrenaline, in general, is involved in processing and control of attention (Selden et al., 1990) by exerting alerting and arousing effects. The noradrenergic system appears to have a role in functionally integrating the brain regions involved in attention, as demonstrated by a study that showed the influence of an alpha2-adrenoceptor-agonist upon the interaction of these regions (Coull et al., 1999). Data from animal studies suggest a role for NA in directing attention towards relevant, salient information (Berridge and Waterhouse, 2003). Consistent with this, NA participates in cognitive flexibility, such as in attentional set-shifting (Kehagia et al., 2010). A study investigating the role of NA in humans found that it modulated attention in a centrally mediated manner, with impairment by the non-selective beta blocker propanolol independent of target valence (De Martino et al., 2008). In addition, modulation of extinction by NA has been reported in various studies on (contextual) extinction learning. Fear extinction in mice and rats was facilitated in a context different from the acquisition context by systemic administration, prior to extinction training, of the alpha-2-receptor antagonist yohimbine, which increases NA release (Cain et al., 2004; Morris and Bouton, 2007). In contrast, systemic administration of the beta-receptor antagonist propanolol impaired retrieval of contextual conditioned fear (Ouyang and Thomas, 2005). Extinction learning of an appetitive response was impaired after NA depletion (Mason and Iversen, 1975, 1978; Mason, 1979), while extinction learning of an instrumental response to a compound of extinguished stimuli was found enhanced by systemic administration of the NA reuptake inhibitor atomoxetine in rats (Janak and Corbit, 2010). In addition, long-term extinction was increased by atomoxetine in rats (Janak et al., 2012).

The role of NA in prefrontal regions for extinction learning has been researched in only a few animal studies. NA release was increased in mPFC of rats exposed to an appetitive extinction paradigm (Mingote et al., 2004). Another study measuring NA release in rat medial PFC during reversal/extinction learning found that it remained constant over conditions (van der Meulen et al., 2007), pointing towards a generally arousing role for NA. Infusions of propanolol into infralimbic medial PFC impaired fear extinction but not consolidation if administered after extinction training (Mueller et al., 2008), suggesting that the crucial role of infralimbic NA is restricted to the extinction learning process proper. Hippocampal NA activation is also a factor in extinction learning. Local infusion of NA into dorsal hippocampus after extinction training enhanced the long term memory for fear extinction (Chai et al., 2014) and extinction retrieval (Rosa et al., 2014), while NA depletion in hippocampal/cortical regions was found correlated with the size of the extinction deficit of a classically conditioned response (McCormick and Thompson, 1982).

Atomoxetine as a selective NA reuptake inhibitor has been shown to modulate attentional processes in various tasks in addition to its effects upon extinction learning. In both mice and rats, atomoxetine increased extracellular levels of NA in PFC (Bymaster et al., 2002; Koda et al., 2010) and in hippocampus (Swanson et al., 2006). With regard to behavioral effects in healthy human subjects, a single dose of atomoxetine has been shown to increase inhibitory control (Chamberlain et al., 2009) as well as heighten phasic alertness, with enhanced neuronal sensitivity for errors that may be due to a more salient representation of the task (Graf et al., 2011). In rats, atomoxetine was able to reverse attentional deficits caused by noradrenergic deafferentiation of the mPFC, while at the same time causing performance deficits in set-shifting in non-lesioned animals (Newman et al., 2008). Thus, atomoxetine appears to be suitable for effectively manipulating prefrontal and hippocampal activation as well as behavior.

In the present study, we aimed to investigate the effects of an NA reuptake inhibitor, in humans upon extinction learning and the renewal effect in an associative learning task. In this task, participants were required to learn relations between cues and outcomes presented in particular contexts, which are reversed during the extinction learning phase. This predictive learning task (Üngör and Lachnit, 2006), which we already used in a previous study (Lissek et al., 2013) features an ABA design previously shown to reliably evoke a renewal effect.

We hypothesized that the enhanced activation of the attentional system in the experimental group will lead to superior extinction learning performance compared to a placebo group. In consequence, we expect a more prominent renewal effect in the experimental group. Moreover, we assume that the brain areas that are active both in extinction and in attentional processing, such as medial PFC, are presumably core regions for exhibiting NA-related effects in extinction learning and the renewal effect. Enhanced recruitment of attentional resources should reflect in increased brain activation in regions processing extinction learning and retrieval decisions.

## Materials and methods

### Participants

Forty healthy right-handed volunteers (20 females, 20 males), mean age 24.89 years +/− 3.16 years st.dev., range 19–31 years, without a history of neurological disorders participated in this study. The participants received a monetary compensation for their participation (in the amount of € 60). Participants were randomly assigned to the experimental atomoxetine (ATO) and placebo control (PLAC) groups. Mean age within the groups was 24.89 years +/− 3.23 st.dev., range 19–30 years in ATO and 24.88 years +/− 3.20 st.dev., range 20–31 years in PLAC. Within these groups, participants were further assigned to either a renewal (REN) group or a no renewal (NOREN) group depending on whether or not they showed a renewal effect during the test phase of the predictive learning task (the procedure is described in detail in Section “Behavioral Data Analysis)”.

### Ethics statement

All subjects participated in this study after giving written informed consent. The protocol was approved by the local Ethics Committee of the Ruhr-University Bochum. The study conforms to the Code of Ethics of the World Medical Association (Declaration of Helsinki). Prior to the experiments, participants received handouts informing them about the functional magnetic resonance imaging (fMRI) procedures and the NA reuptake inhibitor atomoxetine.

### Predictive learning task

The predictive learning task that we used in this study was originally conceived by Üngör and Lachnit (2006) to explore and further illustrate the context-dependency of extinction learning. Its efficiency in evoking a renewal effect was demonstrated in several behavioral studies using this specific design (Üngör and Lachnit, 2006, 2008; Rosas and Callejas-Aguilera, 2006; Nelson and Callejas-Aguilera, 2007; Lucke et al., 2013). We adapted this task for use in an fMRI setting and have used it in a previous fMRI study (Lissek et al., 2013).

**Figure 1:**
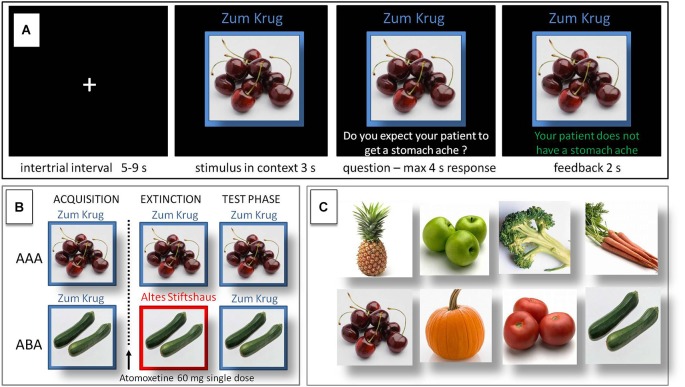
**Predictive learning task. (A)** Example of a trial during acquisition of the task. Participants learned to predict whether certain kinds of food, eaten in a certain restaurant, would cause a stomach ache or not. After an intertrial interval of 5–9 s the stimulus was presented in its context for 3 s, then a question was superimposed on the screen “Will the patient get a stomach ache?” for maximum 4 s response time. Feedback was shown for 2 s, providing the correct answer, e.g., “The patient does not have a stomach ache”. **(B)** Design of the predictive learning task. In condition AAA, extinction occurs in the same context as acquisition. In condition ABA, extinction occurs in a context different from that during acquisition. In both conditions, the final test for the renewal effect is performed in the context of acquisition. **(C)** Food images used as stimuli. Reprinted from Lissek et al. (2013) with permission from Elsevier.

In the predictive learning task, participants were asked to put themselves in the position of a physician and predict whether various articles of food served in different restaurants would lead to the aversive consequence of a stomach ache in their patient. The learning process consisted of the three successive phases of (a) acquisition of associations; (b) extinction learning; and (c) recall phase (see Figure 1). During the acquisition phase (80 trials), participants learned to associate an article of food with a specific consequence. In each trial, one of eight stimuli (vegetables or fruits) was presented to the participant in one of two different contexts (indicated by the restaurant names “Zum Krug” (The Mug) and “Altes Stiftshaus” (The Dome) and a frame in either red or blue color). The stimulus in its context was first presented alone for 3 s, then a question asking whether the patient will develop a stomach ache was superimposed, with the response options “Yes” or “No”. Response time was 4 s, participants responded by pressing the respective button on an fMRI-ready keyboard (Lumitouch, Photon Control Inc. Canada). After the response, or in case of a missing response after expiration of the response time, a feedback with the correct answer was displayed for 2 s, i.e., “The patient has a stomach ache” or “The patient does not have a stomach ache”. The actual response of the participant was not commented upon. The food stimuli were presented in randomized order, each stimulus was presented ten times. Four stimuli were presented per context. Stimuli were counterbalanced with regard to their causing the aversive consequence of a stomach ache, with two stimuli per context causing stomach ache during acquisition, while the other two did not.

During the extinction phase (80 trials), half of the stimuli were presented in the same context as during acquisition (condition AAA—no context change—40 trials) and the other half in the other context (condition ABA—context change—40 trials) in randomized order. In addition, stimuli were subdivided into two types: for actual “extinction stimuli”, the consequence changed and the new consequence had to be learned, for “distractor stimuli”, which were introduced in order to make overall learning more difficult, the consequence remained unchanged. In each context we used two extinction stimuli and two distractor stimuli. In all other respects, trials were identical to those during acquisition.

During the recall phase (40 trials), all stimuli were presented once again in the context of acquisition (five presentations per stimulus). Trials were identical to those during acquisition with the exception that, during the recall phase, no feedback with the correct response was given.

### Procedure

In a first fMRI session, participants performed the acquisition phase of the predictive learning task. After this session, the selective NA reuptake inhibitor atomoxetine was administered orally in a single dose of 60 mg. Control participants received an identical-looking placebo.

After drug administration, participants rested for 90 min. The second fMRI session was performed in a time window of about 90–120 min after administration of the drug. The task timing was based on the phase of peak plasma levels for atomoxetine (60–120 min after oral ingestion in adults) (Sauer et al., 2005; Chamberlain and Robbins, 2013).

### Imaging data acquisition

Functional and structural brain scans were acquired using a whole-body 3T scanner (Philips Achieva 3.0 T X-Series, Philips, The Netherlands) with a 32-channel SENSE head coil. Blood-oxygen level dependent (BOLD) contrast images were obtained with a dynamic T2* weighted gradient echo EPI sequence using SENSE (TR 3200 ms, TE 35 ms, flip angle 90°, field of view 224 mm, slice thickness 3.0 mm, voxel size 2.0 × 2.0 × 3.0 mm). We acquired 45 transaxial slices parallel to the anterior commissure—posterior commissure (AC-PC) line which covered the whole brain. High resolution structural brain scans of each participant were acquired using an isotropic T1 TFE sequence (field of view 240 mm, slice thickness 1.0 mm, voxel size 1 × 1 × 1 mm) with 220 transversally oriented slices covering the whole brain.

The task was presented to the participants via fMRI-ready LCD-goggles (Visuastim Digital, Resonance Technology Inc., Northridge, CA, USA) connected to a laptop which ran specific software programmed in Matlab. Responses were given by means of an fMRI-ready keyboard (Lumitouch response pad, Photon Control Inc., Canada).

### Imaging data analysis

For preprocessing and statistical analysis of fMRI data, we used the software Statistical Parametric Mapping (SPM), Version 8 (Welcome Department of Cognitive Neurology, London, UK), implemented in Matlab 7.6.0 (Mathworks, Natick, MA, USA). Three dummy scans, during which BOLD signal reached steady state, preceded the actual data acquisition of each session, thus preprocessing started with the first acquired volume. Preprocessing at the single subject level consisted of the following steps: slice timing correction to account for time differences due to multislice image acquisition; realignment of all volumes to the first volume for motion correction, spatial normalization into standard stereotactic coordinates with 2 × 2 × 2 mm^3^ using an EPI template of the Montreal Neurological Institute (MNI), smoothing with a 6 mm full-width half-maximum (FWHM) kernel, in accordance with the standard SPM procedure. The acceptable limit for head motion was 2 mm for translational movements and 0.5° for rotational movements.

In a first level single subject analysis we calculated activation during extinction learning and recall phases in the conditions ABA and AAA, respectively. The contrasts were calculated within a combined anatomically defined mask which was constructed using the software MARINA (BION Bender Institute of Neuroimaging, University of Giessen, Germany) (Walter et al., 2003). The mask contained, as *a priori* regions of interest, PFC, hippocampus, amygdala, and insula. All data contained in this combined mask were analyzed together in a single analysis. We used an event-related design, modeling the events of each trial (stimulus and questions presentation, feedback presentation) using distinct stick functions convolved with the default HRF in SPM, with our analysis based on the stimulus presentation phase of each trial. The contrast images from these analyses were entered into second-level random-effects analyses to calculate in one-sample tests the activation patterns of the experimental and control groups for the different contrasts, using a threshold of *p* < 0.05 FWE-corrected (Family-Wise Error) for multiple comparisons with a minimal cluster size (k) of 10 voxels. Moreover, we calculated two-sample tests to directly investigate in which regions the experimental group showed enhanced activation compared to controls. To identify subtle group differences in these extinction-relevant regions in a hypothesis-led anatomically constrained manner, we used a more liberal threshold of *p* < 0.01 uncorrected.

In additional analyses, we evaluated the relation between BOLD signal changes in extinction-relevant brain regions and behavioral data. By means of one-sample tests we identified extinction-relevant ROIs with common activation across groups for contrasts of ABA and AAA trials during extinction learning and recall phase (vmPFC, hippocampus, amygdala, anterior cingulate) and extracted their mean signal intensities (in arbitrary units) using the MarsBar tool (Brett et al., 2002) in SPM 8, in order to perform correlational analyses on potential relations between learning performance and brain activation.

### Behavioral data analysis

For all three learning phases, log files were written that contained information on response latency, response type, and correctness of response for all learning phases. For calculation of the renewal effect, only responses to stimuli with consequence change (extinction stimuli) during the recall phase were analyzed. Based on the literature, the behavioral renewal effect in the predictive learning task should occur only in the condition ABA, in which extinction is performed in a context different from the context present during acquisition and recall phase. During the recall phase, a renewal effect occurs if a response is given that was correct during acquisition, but wrong during extinction (e.g., if in acquisition in context A cherries cause stomach ache, and in extinction learning in context B they no longer cause a stomach ache, then a renewal effect response during recall in context A would be consistent with cherries causing stomach ache). Statistical analyses were performed using the IBM SPSS Statistics for Windows software package, version 20.0 (Armonk, NY:IBM Corp). To test our directional hypotheses regarding performance improvements following the experimental treatment we used one-tailed *t*-tests. In order to evaluate the learning progress during extinction learning, we divided the extinction session into 8 blocks with 10 trials and performed an ANOVA with repeated measures for the factor block. Furthermore, we used *post hoc* tests to separately analyze group differences in performance during: (a) the first block of learning, during which participants experience the surprise event of changed contingencies between stimulus and outcome; (b) early extinction learning, consisting of blocks 2–5; and (c) late extinction learning, consisting of blocks 6–8.

In our previous study using the predictive learning task we found that a considerable portion (about 40%) of the participants did not exhibit the renewal effect. This is a typical finding that also appears in this type of task outside an fMRI setting (Lissek et al., 2013). For further evaluation of their behavioral data, participants were therefore assigned to either the REN (showing renewal) or the NOREN (no renewal) group, respectively. Group assignment was based on their results in the ABA trials with consequence change during the recall phase, i.e., those trials designed to evoke the renewal effect. Participants who never showed a renewal effect (i.e., on any of these trials or 0% renewal responses) were assigned to the NOREN group, whereas participants who showed a renewal effect were assigned to the REN group.

## Results

### Behavioral results

#### Extinction learning

**Figure 2:**
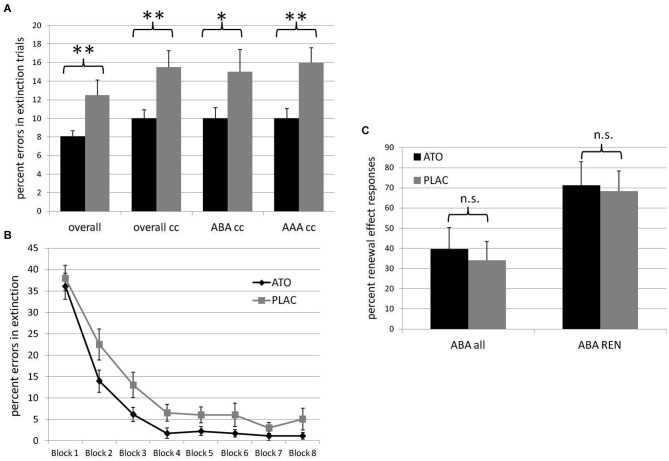
**Behavioral results. (A)** Percentage of errors in extinction trials in the ATO (black) and PLAC (gray) groups. The ATO group made significantly less errors than the PLAC group over all extinction trials (overall), in extinction trials with consequence change only (overall cc), in ABA extinction trials with consequence change (ABA cc) and in AAA extinction trials with consequence change (AAA cc). **(B)** Extinction learning curves for ATO (black) and PLAC (gray) participants, with the extinction session divided into eight blocks of 10 trials each. During initial extinction in block 1, error rates did not differ between groups. However, in subsequent learning, the PLAC group was significantly slower in reducing errors during blocks 2–5. **(C)** Percent renewal effect responses in all participants (ABA all) and only in those participants who actually showed a renewal effect (ABA REN) in the ATO (black) and PLAC (gray) groups. ATO and PLAC groups did not differ with regard to the strength of the renewal effect they exhibited.

As hypothesized, the atomoxetine group, compared to placebo, showed significantly faster extinction learning in terms of the percentage of overall errors (ATO mean 8.055% +/− s.e.m. 0.616, PLAC mean 12.5% +/− s.e.m. 1.619; *t*_(38)_ = 2.460; *p* = 0.0095), errors in trials with consequence change (ATO mean 10.0% +/− s.e.m. 0.904, PLAC mean 15.5% +/− s.e.m. 1.754; *t*_(38)_ = 2.695; *p* = 0.0055), errors in trials with consequence change in an identical context (AAA condition) (ATO mean 10.0% +/− s.e.m. 1.069; PLAC mean 16.0% +/− s.e.m. 1.60; *t*_(38)_ = 3.038; *p* = 0.002), as well as in trials with consequence change in a novel context (ABA condition) (ATO mean 10.0% +/− s.e.m. 1.14, PLAC mean 15.0% +/− s.e.m. 2.43; *t*_(38)_ = 1.794; *p* = 0.0405; See Figure 2A).

In order to evaluate the groups’ learning progress, we divided the extinction session into 8 blocks with 10 trials each and calculated the percentage of overall extinction errors separately for each of these blocks (see Figure 2B). A repeated measures ANOVA showed a significant main effect of the repeated factor block (*F*_(7)_ = 25.978; *p* = 0.000), as well as a significant main effect of group (*F*_(1)_ = 6.244; *p* = 0.017). In both groups, error rates declined across blocks, with no significant interaction (*F*_(7)_ = 0.676; *p* = 0.692). While ATO and PLAC had similar error rates during initial exposure to the changed stimulus-outcome contingencies (first block of extinction learning: ATO 36.1% +/− 3.04 s.e.m.; PLAC 38% +/− 2.96 s.e.m.; *t*_(36)_ = 0.445; *p* = 0.329), during the following early extinction learning phase the PLAC group made significantly more errors than the ATO group (blocks 2ă5: ATO 5.97% +/− 0.86 s.e.m; PLAC 12.0% +/− 2.22 s.e.m.; *t*_(36)_ = 2.424; *p* = 0.009). During later extinction learning, a performance difference persisted, but was no longer significant (blocks 6–8: ATO 1.48% +/− 0.77 s.e.m.; PLAC 4.67% +/− 1.81 s.e.m.; *t*_(36)_ = 1.614; *p* = 0.0595) (All *t*-tests one-tailed).

The higher variability in PLAC during later extinction learning was due to three participants out of 20 showing an error level of 20–26% in these blocks, while the error level of the majority was 0–6.6%. The ATO group’s lower variability during this phase was due to only two participants out of 20 showing 10–13% errors and the remaining 18 participants 0–3% errors.

ATO and PLAC groups did not differ in their response times during extinction learning (ATO mean 0.5423 s +/− 0.045 s.e.m, PLAC mean 0.6493 s +/− 0.050 s.e.m. *t*_(38)_ = 1.547; *p* = 0.131 two-tailed).

Also, ATO and PLAC groups did not differ in pre-treatment acquisition of the original cue-outcome associations in terms of the percentage of errors made during acquisition (ATO mean 12.31% +/− s.e.m. 1.926, PLAC mean 13.33% +/− s.e.m. 1.928; *t*_(38)_ = 0.373; *p* = 0.711 two-tailed).

#### Renewal effect

Stimulation of the noradrenergic system did not significantly enhance the renewal effect: we observed no significant differences between the atomoxetine and the placebo group with regard to the strength of the renewal effect, i.e., the preferred recall of associations correct for the acquisition phase in recall trials where extinction occurred in a different context. The PLAC group showed 34.16% (+/− s.e.m 9.246) renewal responses in the ABA condition, compared to the ATO group with 39.63% (+/− s.e.m. 10.629; *t*_(38)_ = 0.390; *p* = 0.345). In recall trials for which previous extinction occurred in an identical context (AAA condition), ATO participants were significantly better in avoiding errors, instead responding with associations that were correct during extinction (ATO mean 0.00% errors +/− s.e.m. 0.000, PLAC mean 4.16% errors +/− s.e.m. 2.049; *t*_(38)_ = 1.925; *p* = 0.031; See Figure 2C).

#### Gender differences

We did not observe any significant gender differences within the ATO group, which comprised nine womens and nine mens, neither in the error rate during acquisition (*t*_(16)_ = 0.677; *p* = 0.508) and extinction learning (*t*_(16)_ = −0.918; *p* = 0.372) nor in the strength of the renewal effect (*t*_(16)_ = 0.161; *p* = 0.874).

In the PLAC group, which comprised 8 men and 10 women, there was a slight, albeit insignificant, tendency towards a difference in acquisition (*t*_(16)_ = −1.920; *p* = 0.059), but not in extinction learning (*t*_(16)_ = −1.656; *p* = 0.117) or in the strength of the renewal effect (*t*_(16)_ = 0.026; *p* = 0.979).

Across groups, there were no significant differences between men in the ATO and PLAC groups on the one hand (acquisition (*t*_(15)_ = 0.863; *p* = 0.402), extinction (*t*_(16)_ = −1.343; *p* = 0.202), level of renewal (*t*_(16)_ = 0.558; *p* = 0.585)) nor between women in the ATO and PLAC groups on the other (acquisition (*t*_(17)_ = −1.804; *p* = 0.092), extinction learning (*t*_(17)_ = −1.809; *p* = 0.088), level of renewal (*t*_(17)_ = 0.443; *p* = 0.663)).

#### Performance of participants who showed/did not show the renewal effect

In both groups, those participants who showed or did not show the renewal effect were equally distributed: in the ATO group 55.55% of participants showed renewal (REN), 44.45% did not (NOREN) (*χ*^2^ = 0.200; *p* = 0.655), in the PLAC group 50% showed renewal and 50% did not (*χ*^2^ = 0.000; *p* = 1.00). There was no significant difference with regard to the strength of the renewal effect between the REN subgroups, with the ATO REN group exhibiting a mean of 71.33% renewal responses (+/− s.e.m. 11.527) and the PLAC REN group a mean of 68.33% renewal responses (+/− s.e.m. 10.077), *t*_(18)_ = 0.196; *p* = 0.847 two-tailed.

Comparing the extinction learning performance of the REN subgroups yielded a pattern of results similar to that of the complete groups. The percentage of errors in trials with consequence change was significantly lower in the ATO REN group (mean 10.25% +/− s.e.m. 0.946) than in the PLAC REN group (mean 15.25 +/− s.e.m. 2.218); *t*_(18)_ = 2.073; *p* = 0.0.026, as were errors in trials with consequence change in an identical context (AAA condition) (ATO REN mean 10.0% +/− s.e.m. 1.291, PLAC REN mean 16.0% +/− s.e.m. 2.56) *t*_(18)_ = 2.092; *p* = 0.0255.

The performance difference between ATO and PLAC was also present in the subgroups of NOREN partipants. The percentage of errors in all trials was lower in ATO (7.34% +/− s.e.m. 0.763) than in PLAC (11.62% +/+ s.e.m. 1.863; *t*_(16)_ = 1.943; *p* = 0.0035); also the percentage of errors in trials with consequence change was lower in ATO (9.68% +/− s.e.m. 1.731) than in PLAC (15.75% +/− s.e.m. 2.839; *t*_(16)_ = 1.823; *p* = 0.0445). In particular, errors in trials with consequence change in an identical context (AAA) were lower in ATO 10% +/− s.e.m. 1.889) than in PLAC (16% +/− s.em. 2.082; *t*_(16)_ = 2.143; *p* = 0.0245).

### Imaging results

In one-sample tests, we analyzed brain activation during extinction learning and recall phases in the familiar or a novel context for the ATO and PLAC groups separately. In two-sample tests, we directly compared activation patterns in the ATO and PLAC groups.

#### Brain activation during extinction learning and recall in ATO and PLAC groups separately

During extinction learning in a novel context (condition ABA), both groups activated extended regions in bilateral dlPFC (Brodmann Area (BA) 8, 9 and 46) and bilateral OFC (BA 10, 47). In posterior hippocampus and superior temporal regions (BA 38, 22), activation was restricted to the left hemisphere in ATO and bilateral in PLAC. In fusiform gyrus (BA 37), ATO showed small clusters of bilateral activation compared to PLAC with a larger cluster of right-hemispheric activation. In addition, the PLAC group exhibited a more extensive activation of bilateral insula compared to ATO, as well as activations in bilateral precuneus, right-hemispheric cingulate gyrus (BA 32) and bilateral lingual gyrus (BA 19) which were absent in ATO. In contrast to PLAC, the ATO group also showed activation in right-hemispheric amygdala.

During extinction learning in a familiar context (condition AAA), both groups showed prominent activation in bilateral superior temporal (BA 22, 38) and fusiform gyri (BA 37), in bilateral OFC (BA 10, 47) and bilateral hippocampus. In addition, only the ATO group showed participation of Broca’s area (BA 45), while PLAC activated the corresponding right-hemispheric region (BA 44). Also, lingual gyrus (BA 19) activation was observed only in ATO. In contrast, only the PLAC group showed participation of bilateral precuneus and a substantially larger activation in bilateral insula—similar to the pattern exhibited during extinction learning in the novel context.

During recall of stimulus-response associations extinguished in a novel context (condition ABA), both groups show participation of bilateral insula, Broca’s area (BA 44), regions in righthemispheric OFC (ATO BA 10, PLAC BA 47), hippocampus (bilateral in ATO, right-hemispheric in PLAC) and fusiform gyrus (right-hemispheric in ATO, bilateral in PLAC). In addition, only the PLAC group shows activation in bilateral dlPFC (BA 9), right-hemispheric cingulate gyrus (BA 32), and right-hemispheric lingual gyrus.

During recall of associations extinguished in an identical context (condition AAA), both groups reveal participation of several clusters in bilateral dlPFC (BA 8, 9, 46), bilateral hippocampus, fusiform, and lingual gyri. Activation in cingulate regions was bilateral in ATO and exclusively left-hemispheric in PLAC, in OFC left-hemispheric in ATO and right-hemispheric in PLAC, in superior temporal regions bilateral in ATO and right-hemispheric in PLAC. Only the ATO group showed activation in Broca’s area (BA 44) during this phase (see Table 1).

**Table 1:** **One-sample tests of ATO and PLAC groups, FWE *p* < 0.05 *k* = 10**.

Area	BA	Hem	Extinction ABA						Extinction AAA					
			ATO			PLAC			ATO			PLAC		
			MNI	***t***-value	Voxel	MNI	***t***-value	Voxel	MNI	***t*-value**	Voxel	MNI	***t***-value	Voxel
dlPFC	9	L				−34 32 44	5.30	20
		R	44 35 35	5.93	42	22 42 40	7.33	120
	8	L	−34 22 50	5.74	55	−30 18 50	7.47	78
		R	44 16 46	6.53	49	42 22 48	9.34	127
	46	R	50 44 14	5.52	19	45 40 28	8.93	95				22 50 20	7.52	28
												38 26 28	5.95	38
Broca’s area	45	L							−54 20 6	5.32	15
BA 44		R										56 10 8	5.54	18
OFC	47	L	−18 14 −20	6.19	24	−24 12 −16	6.23	24				−42 20 −12	7.95	101
			−42 18 −10	5.60	40
		R	44 38 −10	5.94	13	30 24 −6	6.75	163	48 20 −6	6.09	74
			58 16 −2	5.64	18				47 42 −13	6.13	33
	10	L	−36 54 18	6.69	43	−40 54 8	5.91	15				−38 54 14	9.64	68
		R	42 48 26	7.27	59	45 52 −2	7.24	216	26 58 28	6.54	27	36 52 2	6.21	64
												40 46 18	5.87	77
Cingulate gyrus	32	R				12 40 14	5.58	20
Hippocampus
Posterior		L	−20 −28 −10	5.72	35	−20 −30 −4	7.44	28				−22 −32 −6	8.62	75
			−24 −30 −6	6.63	76
		R				20 −40 −4	5.49	81	24 −34 0	6.52	55	20 −29 −6	9.52	114
Mid		R				32 −18 −12	5.54	81	−28 −24 −12	8.37	53
Parahippocampal g.	27	L	−16 −36 −6	4.75	35
		R				15 −28 −4	9.01	121	22 −34 −10	6.51	45
Lingual gyrus	19	L				−15 −50 −8	7.30	144	10 −35 −2	7.11	60
						−8 −35 −4	5.40	20
		R				14 −48 −2	5.14	114
Amygdala		R	26 2 −14	5.47	13							28 0 −12	5.87	24
Insula		L	−38 0 6	6.69	58	−42 15 −4	5.68	62	−40 2 0	5.24	28	−44 −4 0	7.27	533
						−40 −4 −8	5.78	16
						−42 −8 8	6.37	71
		R				36 14 −4	6.7	57				40 16 −8	5.45	78
Superior temporal g.	38	L							−52 12 −8	7.25	24
		R				54 20 −8	8.79	74	56 14 −10	6.43	54	54 18 −10	7.22	43
	22	L	−58 8 −2	5.30	99	−56 12 −6	7.48	36	−50 2 −2	6.07	42	−55 12 −6	6.08	58
		R										54 10 −4	7.14	56
Fusiform gyrus	37	L	−38 −46 −18	5.84	11				−22 −44 −18	6.11	38	−22 −48 −16	7.02	164
	37	R	20 −50 −14	5.08	10	32 −52 −14	6.54	78	30 −35 −25	6.70	125	36 −52 −14	7.30	86
Transverse temp. g.	41	L				−50 −18 12	7.15	13
Precuneus		L				−4 −48 68	6.59	103				−16 −44 72	7.56	129
		R				4 −46 68	6.89	40				2 −46 70	6.55	39
dlPFC	9	L				−52 4 24	5.67	31	−56 6 34	6.55	48	−58 8 32	6.63	67
						−38 26 32	7.22	20
		R				38 46 32	6.29	39	62 8 26	6.37	33	44 40 30	7.05	14
									50 34 30	5.67	21	50 4 42	6.54	57
	8	R							52 12 48	5.11	17	56 10 42	6.44	24
	46	L							−48 42 14	5.23	25
		R										52 28 16	5.20	18
	45	R							60 26 16	6.59	39
OFC	47	L							−34 26 −6	5.75	49
		R				34 18 −2	8.71	68				40 16 −6	6.21	18
	10	R	40 50 14	5.68	10
			34 54 30	6.48	21
Broca’s area	44	L	−54 6 18	6.02	35	−58 6 20	6.67	42	−58 10 12	5.39	60
Cingulate gyrus	31	L							−6 −26 46	5.89	21
		R							10 −30 44	6.58	41
	32	L										−4 6 46	6.39	106
		R				10 18 30	7.22	234
	24	L										−4 2 50	6.40	39
Hippocampus
Posterior		L	−22 −30 −4	7.21	16				−20 −30 −4	6.13	19	−24 −30 −4	6.07	10
		R	24 −28 −6	5.04	10	24 −30 −4	7.89	43	22 −28 −6	6.30	70	18 −30 −4	5.95	10
Insula	13	R	40 12 −2	5.99	18	45 8 0	7.59	56	32 22 −10	6.17	16	32 20 4	5.94	14
									36 8 0	5.55	12	42 20 −6	6.41	59
		L	−40 14 2	5.35	34	−40 12 −2	6.83	37	−26 24 −6	7.39	88
			−36 0 −2	6.09	10	−38 −2 10	7.62	26	−36 −2 0	6.80	51
			−38 −2 14	6.83	36				−40 −16 14	5.04	50
			−48 −22 16	7.04	55
Superior temporal g.	22	L	−56 12 2	5.90	26				−50 2 0	6.73	62	52 15 −10	5.72	11
		R							52 14 −6	5.21	14
Fusiform gyrus	37	L				−26 −50 −12	7.89	67	−26 −50 −12	6.07	64	−30 −46 −18	6.55	65
		R	30 −50 −14	7.42	45	32 −52 −14	9.93	117	26 −48 −16	10.05	146	28 −50 −14	9.66	209
Lingual gyrus	19	L							−16 −50 −6	8.23	70	−20 −50 −10	6.51	26
		R				20 −44 −8	6.45	48	18 −42 −2	6.10	44	16 −48 −8	6.86	86

#### Effects of NA on brain activation during extinction learning—compared to placebo

During extinction learning in the AAA condition, ATO participants compared to PLAC show significantly higher activation in bilateral anterior and posterior hippocampus, bilateral insula (BA 13), orbitofrontal cortex (BA 47 and 10), left vmPFC (BA 10) and ACC/cingulate gyrus (BA 24, 32), in left superior temporal gyrus (BA 22, 38, 41) as well as in bilateral amygdala. During extinction learning in the ABA condition, ATO exhibits increased activation compared to PLAC in right dlPFC (BA 46), right vmPFC (BA 10), right hippocampus, bilateral ACC (BA 32) as well as right insula.

Areas where ATO showed small clusters of lower activation than PLAC were bilateral dlPFC (BA 8) and vmPFC (BA 10/11) as well as left insula (BA 13) in ABA extinction. During AAA extinction, there was no region in which ATO exhibited lower activation than PLAC.

#### Effects of NA on brain activation during extinction recall—compared to placebo

During recall in the AAA condition, the ATO group exhibits higher activation than PLAC in right dlPFC (BA 46), bilateral vmPFC (BA 10,11), right hippocampus, bilateral ACC (BA 32,25), right insula (BA 13) and bilateral superior temporal gyrus (BA 22,41). During recall in the ABA condition, ATO compared to PLAC activated bilateral dlPFC (BA 9), left hippocampus, bilateral ACC (BA 32) and righthemispheric insula.

**Figure 3:**
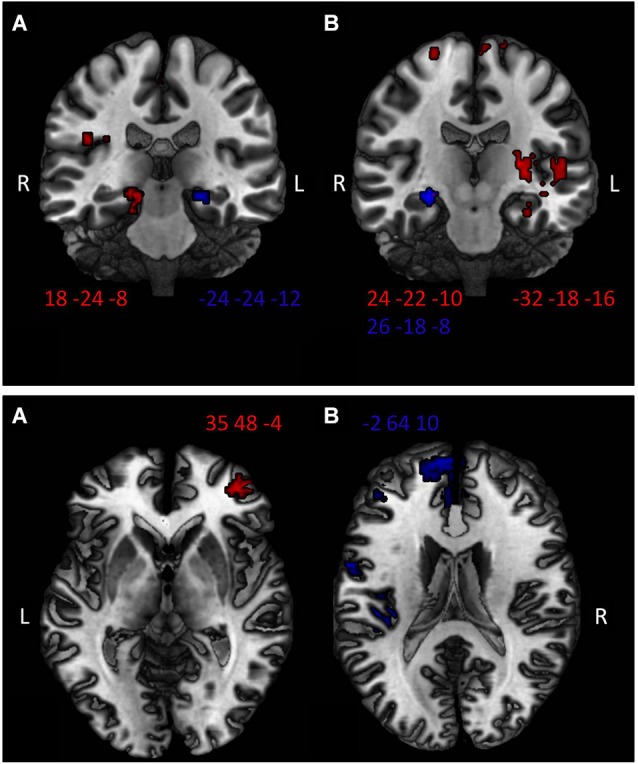
**Top: Higher hippocampal activation in the ATO group compared to PLAC in the ABA condition (panel A) and the AAA condition (panel B) during extinction learning (red) and recall phase (blue)**. Two-sample *t*-tests ATO < PLAC thresholded at *p* < 0.01, minimum cluster size *k* = 10 voxel. Bottom: Higher vmPFC activation of ATO compared to PLAC in **(A)** extinction in a novel context (ABA condition)—righthemispheric BA 10, peak MNI coordinate 35 48 −4; and in **(B)** extinction in the familiar context (AAA condition)—lefthemispheric BA 10, peak MNI coordinate −2 64 10. Two-sample *t*-tests ATO < PLAC thresholded at *p* < 0.01.

In ABA recall, ATO showed lower activation than PLAC in Broca’s Area (BA 45) and a small cluster in left anterior hippocampus. In AAA recall, there was lower activation in ATO in regions in left anterior and right posterior hippocampus as well as in bilateral dlPFC (BA 9) (See Table 2; Figure 3).

**Table 2:** **Two-sample tests comparing performance of ATO and PLAC groups, *p* < 0.01 *k* = 10**.

ATO > PLAC			ABA						AAA					
			Extinction			Recall			Extinction			Recall		
Brain region	BA	Hem	MNI coord	***t***-value	Voxel	MNI coord	***t***-value	Voxel	MNI coord	*t*-value	Voxel	MNI coord	***t*-value**	Voxel
Prefrontal cortex
dlPFC	46	R	52 28 16	3.52	293							42 26 16	3.33	42
	9	L				−34 26 24	4.37	70
		R				36 46 36	3.92	29
OFC	47	L							−28 14 −12	3.46	60
		R							32 16 −20	3.35	80
	10	L							−38 46 6	3.87	96
vmPFC	10	L							−2 64 10	3.98	76	0 64 10	3.25	16
		R	40 48 −4	4.24	70
	11	R										28 56 −8	3.77	13
	44	R							56 4 10	4.79	22
Hippocampus		L				−24 −24 −12	2.95	19	−32 −18 −16	2.83	39
									−36 −16 −12	2.92	12
									−26 −26 −12	3.29	21
		R	18 −24 −8	3.36	15				24 −22 −10	3.13	10	26 −18 −8	3.80	28
									20 −30 −4	2.74	10
Amygdala		L							−28 −6 −16	3.25	23
		R							28 4 −18	3.57	27
Anterior cingulate	32	L	−18 24 30	4.23	83	−10 16 40	2.93	161	−6 44 8	3.56	111	−4 46 15	2.77	42
		R	22 28 24	4.20	92	4 20 30	2.78	11
	24	R							6 22 24	3.21	13
	25	R				2 8 −6	3.38	54				6 12 −10	3.12	13
Cingulate gyrus	24	L							−14 −2 46	3.62	15
		R
Posterior cingulate		L							−10 −44 10	3.09	11
Insula	13	L							−40 -10 6	3.53	154
		R	44 −28 24	3.19	68	40 24 2	3.66	50	34 12 −18	3.69	58	42 −4 0	3.46	26
			30 28 4	3.34	17				34 −26 10	4.12	29
Superior temporal gyrus	22	L							−50 −18 0	3.69	260
	38	L							−54 4 −12	3.44	62
		R										30 16 −30	3.76	54
	41	L							−36 −46 10	4.83	25	−36 −32 10	3.49	13
Prefrontal cortex
dlPFC	8	L	−20 38 50	2.95	10
		R	16 50 46	3.29	18
	9	L										−12 40 32	3.80	35
		R										18 36 34	4.70	49
vmPFC	10	L	−8 62 18	2.82	11
	11	R	26 36 −12	3.43	21
Brocas Area	45	L				−56 20 6	4.00	35
Hippocampus		L				−12 −12 −22	3.34	6(10				−10 −6 −16	3.33	20
		R										28 −40 4	3.43	65
Insula	13	L	−28 14 −10	3.62	17

### Correlations between brain activation and performance

To determine whether activation in particular brain regions was related to performance in extinction and recall, we performed across groups analyses correlating performance data with activation to the stimuli in several task-relevant ROIs derived from onesample tests comprising all participants.

Activation during extinction learning of stimuli in a novel context (ABA) correlated significantly with later recall performance in the following brain regions: In left vmPFC, BA 10 (peak MNI coordinate −12 62 20) there was a positive correlation with the number of renewal effect responses during recall (*r* = 0.326; *p* = 0.040 two-tailed). Thus, the more active the vmPFC was during encoding of the new association in a novel context, the better the assignment of the association to its context in recall. In left anterior hippocampus (peak MNI coordinate −20 −4 −24), we found a negative correlation with the number of renewal responses during recall (*r* = −0.414; *p* = 0.008 two-tailed), suggesting that processes in left anterior hippocampus in these extinction trials were associated to a lower tendency of showing a renewal effect across all participants. Moreover, activity in right anterior cingulate BA 32 (peak MNI coordinate 2 40 12) was negatively correlated with the number of errors in recall of AAA trials (*r* = −0.409; *p* = 0.009 two-tailed), i.e., a low cingulate activation during extinction learning in ABA trials was linked to more incorrect recall of associations in AAA trials. Activation during extinction learning of stimuli in the identical context (AAA) did not correlate significantly with recall performance in the recall phase. During recall of stimuli previously extinguished in the identical context (AAA condition), activation in right amygdala showed a significant negative correlation with the number of recall errors in AAA trials (peak MNI coordinate 32 0 −22) (*r* = −0.405; *p* = 0.009 two-tailed).

## Discussion

### Activation of the noradrenergic system enhances extinction learning while increasing activity in several task-relevant regions

As hypothesized, an NA reuptake inhibitor significantly accelerated extinction learning in the ATO group compared to the PLAC group. Faster extinction learning occurred in both conditions ABA and AAA, suggesting that reversing an established cue-outcome association was easier for ATO participants, regardless of whether stimuli were presented in an identical or a novel context. Overall, this result suggests a higher potential for behavioral flexibility in the ATO group. Our findings correspond to results from animal studies that found instrumental extinction and long-term extinction enhanced by systemic administration of atomoxetine in rats (Janak and Corbit, 2010; Janak et al., 2012). In humans, the effect may well be based on the ability of atomoxetine to increase inhibitory control (Chamberlain et al., 2009), thus enabling a more efficient inhibition of obsolete associations and the respective responses. Moreover, atomoxetine has been shown to heighten phasic alertness in humans (Graf et al., 2011)—a general effect of the agonist which may provide a more salient representation of the task as well as enhanced error sensitivity.

Faster extinction learning in the ATO group was associated with increased activation in distinct hippocampal regions during both the ABA and the AAA condition, i.e., during extinction in a novel as well as in a familiar context, ATO participants showed higher activity in hippocampus than PLAC participants. The present results are consistent with the conclusions from our previous study with the same predictive learning task (Lissek et al., 2013), stating that hippocampus encodes the relation between context and cue-outcome. Here, the stronger activation is presumably related to the ATO group’s more efficient encoding of this relation, which in turn supported their faster extinction learning.

Moreover, corresponding to our hypothesis, the ATO group showed higher activation than PLAC in vmPFC during ABA and AAA extinction. This result suggests that, complementing our findings in the previous study of vmPFC participation in recall of extinction memory, the region also has a prominent role in extinction learning, which is presumably related to quickly adapting response decisions to the changed circumstances, taking context into consideration. The correlation between left vmPFC activity during ABA extinction and the number of renewal effect responses in ABA recall that we observed across groups delivers further evidence for a crucial participation of vmPFC during updating of the relation between cue-outcome association and context which enables a better assignment of an association to its context during recall. A study of human fear extinction learning similarly reported context-dependent activation in hippocampus and vmPFC, suggesting that hippocampus confers context dependance upon vmPFC (Kalisch et al., 2006), which implies that both structures participate in encoding and decoding of the relation between a context, a cue and an outcome. In our study, both vmPFC and hippocampus therefore appear to participate in contextual extinction learning, with hippocampus encoding context and vmPFC decoding this information in order to update and adapt responses appropriately.

As opposed to this pronounced increase we observed a complementary reduction of activation in small clusters of vmPFC during ABA extinction. The left-hemispheric region in which ATO shows lower activation than PLAC largely corresponds to the region in which we found the above-mentioned correlation with the number of renewal responses during ABA recall, which might reflect the slightly, though not significantly, lower percentage of renewal responses in the ATO group.

Other regions that showed higher activation in the ATO group during extinction learning presumably also support facilitated extinction learning, such as distinct regions in insula and anterior cingulate that were more active in both the ABA and AAA condition. Anterior cingulate and anterior insula together have been suggested to constitute a salience detecting network (Sridharan et al., 2008; Vincent et al., 2008; Menon and Uddin, 2010; Craigmyle, 2013). In the context of an overall role of insula in processing salience (Menon and Uddin, 2010), activation of the region in the processing of feedback (regardless of valence) has been found (Bischoff-Grethe et al., 2009). Moreover a function in support of coordination and evaluation of performance in tasks with varying demands has been proposed (Eckert et al., 2009). Moreover, processing of changing reinforcement contingencies (D’Cruz et al., 2011) and error awareness (Ullsperger et al., 2010) have been demonstrated as insula functions. In our previous study (Lissek et al., 2013), insula activation was stronger to the novel context-cue compound, consistent to the proposed function of detecting salience. Within the individual groups as analyzed in the one-sample tests, this effect was observed here too. However, in the direct comparison between groups, ATO exhibits relatively higher insula and anterior cingulate activation for both the ABA and AAA conditions. Thus, an NA reuptake inhibitor enhanced the response of these regions to both novel and familiar context-cue compounds, conceivably in the context of registering errors, an activity which might be related to the ATO group’s lower error rate in both conditions. Increased activation of insula with atomoxetine was previously found associated with successful response inhibition in a stop-signal task (Chamberlain et al., 2009). Higher activation of anterior cingulate regions with atomoxetine compared to placebo was observed also during response inhibition in a go/no-go task (Nandam et al., 2014), suggesting that higher activation in these regions in the ATO group during extinction learning may be related to their superior response inhibition.

In addition, right anterior cingulate activation in ABA extinction correlated with enhanced AAA recall across groups, with higher activation during ABA trials linked to fewer errors in recall of AAA trials; an effect that appears to be related to the significantly better performance of ATO in AAA recall. It can be speculated that during extinction learning in ABA trials, cingulate attentional processing highlighted the importance of context for the change in stimulus-outcome associations, information that could be utilized later for correct responding in trials where the context did not change. However, it remains unclear why such an information transfer did not occur for ABA trials, since there was no significant correlation with performance in ABA recall. A more direct contribution to superior performance in AAA recall was delivered by the right amygdala, which appears to be involved in processing recall of an altered association within an identical context, as higher amygdala activation was associated with fewer errors in AAA recall.

### Activation of the noradrenergic system does not affect the strength of the renewal effect, but is associated with increased activity in regions involved in renewal

Contrary to our hypothesis, the NA reuptake inhibitor atomoxetine did not increase the percentage of renewal effect responses in the ABA condition relative to placebo. Conceivably, also the slower extinction learning progress in PLAC participants yielded a stable level of extinction memory sufficient for producing a comparable renewal effect in both groups. Moreover, the NA agonism-induced superior extinction learning performance does not appear to influence the decision required during the recall phase in the ABA condition, i.e., deciding whether to take context into account in selecting a response.

The behavioral similarities in renewal performance of ATO and PLAC participants partially reflect in brain activation patterns. For example, we observed no differential activation in ventromedial PFC between ATO and PLAC during ABA recall. This area was previously (Lissek et al., 2013) found more active in retrieval of context-cue associations of the ABA condition in those participants who actually displayed a renewal effect compared to those who did not. Therefore, high vmPFC activation in ABA recall is apparently related to the decision to consider context in responding. Here, the lack of differential vmPFC activation for ABA recall in the direct comparison of ATO and PLAC groups may thus correspond to the finding that the groups show a similar level of renewal, and that the NA reuptake inhibitor does not affect processing of renewal.

In contrast, activation during recall in another region that is relevant for processing contextual information in extinction (i.e., the hippocampus) differs between the groups. ATO compared to PLAC shows higher activation in a region in left mid hippocampus in response to ABA trials, and in right mid hippocampus in AAA trials. On the other hand, ATO exhibits reduced activation in left anterior hippocampus in ABA and AAA recall and additionally in right posterior hippocampus in AAA recall. The activation level in left anterior hippocampus appears relevant for renewal only during ABA extinction learning, where across groups it correlated negatively with the strength of the renewal effect exhibited in ABA recall, but not during recall proper, since the differential recall activation in this region did not affect the strength of renewal. Thus, higher respectively lower hippocampal activation appears unrelated to the level of the renewal effect displayed by the groups, which corresponds to the result of our previous study of no differential hippocampal activation in REN and NOREN groups during ABA recall. Overall, these findings support our assumption that prominent hippocampal activation is crucial during extinction learning by marking relevance of context, but that during extinction recall it acts in concert with vmPFC activation to produce a renewal effect.

Further regions in which ATO showed higher activation than PLAC during ABA recall were bilateral cingulate (BA 24) as well as bilateral dlPFC (BA 9). None of these regions alone or in concert had an impact on the strength of the renewal effect. This conclusion corresponds to the finding that this observed activation pattern actually constitutes a subset of the pattern exhibited during ABA extinction recall by participants who did not show a renewal effect (Lissek et al., 2013). There, among a number of other regions, we too found higher activation in cingulate gyrus (BA 24), right dlPFC (BA 9) and right lateral OFC (BA 10). Therefore, the activation of those regions does not appear to contribute to generating a renewal effect.

## Conclusion

To the best of our knowledge, this study is the first to demonstrate the effects of NA stimulation upon brain activation patterns associated with extinction learning and the renewal effect of extinction in healthy human participants.

In summary, our results deliver evidence for the involvement of the human noradrenergic system in the modification of established associations during extinction learning, which is presumably related to its attention-enhancing functions on the one hand and its role in response inhibition, which is associated with insula and anterior cingulate activity, on the other. Moreover, NA-induced activity increases in hippocampus and vmPFC contributed to more efficient encoding of the relationship between context and cue-outcome. By means of this orchestration, the noradrenergic system appears to support behavioral flexibility in extinction learning which enables swift reversals of established cue-outcome associations. In contrast, assignment of an association to a context and subsequent decision on the adequate response in recall of extinction memories, as exemplified in the renewal effect, presumably operate largely independently of noradrenergic mechanisms.

## Conflict of interest statement

The authors declare that the research was conducted in the absence of any commercial or financial relationships that could be construed as a potential conflict of interest.
